# Cannabinoid CB_1_ Receptors Mediate the Gastroprotective Effect of Neurotensin

**Published:** 2012

**Authors:** Parichehr Hassanzadeh, Elham Arbabi

**Affiliations:** 1 *Research Centre for Gastroenterology and Liver Diseases, Shahid Beheshti University of Medical Sciences, Tehran, Iran*

**Keywords:** CB1 receptors, Gastroprotective action, Neurotensin

## Abstract

**Objective(s):**

Several lines of evidence indicate that neuropeptides exhibit protective properties against gastroduodenal ulcers. Neurotensin, a gut-brain neuropeptide, is implicated in a number of physiological processes in the central nervous system and peripheral tissues including gastrointestinal tract. In the present study, we aimed to investigate the gastroprotective potential of either peripherally or centrally administered neurotensin with a look at the role of the cannabinoid CB_1_ receptors which are located in brain areas implicated in the regulation of gastric functions.

**Materials and Methods:**

Gastric mucosal damage was induced by intragastric administration of acidified ethanol in male Wistar rats. One hour later, gastric lesions were evaluated macroscopically. In gastroprotection study, neurotensin was administered either intravenously (1.5, 3, and 5 µM/kg) or intracerebroventricularly (0.5, 1, and 2.5 nM/rat) 30 min before the ethanol challenge. In order to evaluate the involvement of central CB_1_ receptors in the gastroprotective effect of neurotensin, the CB_1_ receptor antagonist AM251 (5, 10, and 15 nM/rat) was given i.c.v. 30 min prior to the administration of neurotensin. The effects of AM251 on the intact stomach and ethanol-induced gastric lesions were also evaluated.

**Results:**

Acidified ethanol induced large areas of gastric lesions which were significantly reduced by the highest dose of neurotensin in i.v. or i.c.v. application. The gastroprotective effect of neurotensin was prevented by pretreatment with 15 nM/rat AM251. AM251 had no effect by itself.

**Conclusion:**

Peripherally or centrally given neurotensin protects gastric mucosa against damage induced by acidified ethanol through the activation of central cannabinoid CB_1 _receptors.

## Introduction

Peptic ulcer disease refers to painful sores or ulcers in the lining of the stomach or the first part of the small intestine, the duodenum. Acid and pepsin play a prominent role in the development of peptic ulcer disease (1). Antisecretory drugs including proton pump inhibitors and histamine H_2_-receptor blocking drugs have emerged as effective therapeutic agents (2, 3). However, following the introduction of proton pump inhibitors, bleeding complications and fatal events have not been decreased. According to the reports, gastric bleeding associated with stress ulcer occurs even in intensive care units leading to a remarkable mortality (4-6). 

In general, acid secretion is normal or even decreased in upper gastric ulcers (7) indicating that decreased mucosal resistance may be responsible for the development of mucosal damage or impaired mucosal defensive mechanisms. As it is acknowledged, mucosal defence may be initiated both peripherally and centrally. There are structural and functional elements which constitute the peripheral mediators involved in mucosal protection and integrity such as the adherent mucus HCO_3_^–^ layer and gastric mucosal blood flow (8). Meanwhile, in contrast to the peripheral mechanisms of mucosal protection, much less attention has been paid to the central mediators or brain area(s) which may be implicated in the maintenance of gastric mucosal integrity and/or stimulation of mucosal defensive mechanisms. According to the reports, brain areas such as dorsal vagal complex as well as several neuropeptides are involved in the maintenance of gastric functions including acid secretion, motility, and gastric mucosal defence (9, 10). 

Neuropeptides are small molecules produced by a variety of cells including neurons and immune cells and modulate several biological processes such as the motility, electrolyte transport, mucosal blood flow and cell growth (11). Neuropeptides released during inflammatory conditions are able to modify the activity of cells responsible either to trigger tissue damage or promote healing. Neuropeptides have also been shown to influence gastric functions (12, 13). In recent years, neurotensin, a gut-brain neuropeptide, has attracted growing interest. This tridecapeptide which was originally isolated from hypothalamus (14), is widely distributed throughout the nervous system and is responsible for many characteristics of a classical neurotransmitter or circulating hormone (15). Neurotensin is synthesized in small amounts in the brain and in relatively large quantities throughout the gastrointestinal tract (GIT) (16). Since its isolation, identification and synthesis, neurotensin has been shown to be implicated in a number of physiological processes such as vasodilatation, nociception and secretory or motor effects on the mammalian GIT (17-20). In addition to its neuromodulatory effect, neurotensin facilitates the repair of the wounded epithelium that may lead to the beneficial effects in chronic models of colitis (21). Moreover, neurotensin interacts with leukocytes, mast cells and macrophages and modulates immune responses, epithelial cell proliferation and chloride secretion (22-26). 

This background prompted us to evaluate the gastroprotective potential of neurotensin following either peripheral or central administration. Moreover, as a mechanistic approach, we looked at the role of the cannabinoid CB_1_ receptors in the potential gastroprotective effect of neurotensin. According to the reports, the endocannabinoid system plays an important role in the GIT under physiological or pathophysiological conditions (27, 28). In addition, cannabinoid CB_1_ receptors are present in the enteric nervous system and dorsal vagal complex of the brain stem, an area implicated in the regulation of gastric functions (29). 

## Materials and Methods

***Animals***

Male Wistar rats weighing 220-250 g obtained from Pasteur Institute of Iran were used in this study. Animals were kept in a 12 hr light/dark cycle under controlled temperature and humidity and fasted for 24 hr but had free access to water until 4 hr before the experiment. All experimental procedures were approved by the Local Ethics Committee. 

***Induction of gastric mucosal damage***

Twenty four hr after food deprivation, animals received 1 ml acidified ethanol (98% ethanol in 200 mM/l HCl, pH 6.7) intragastrically via a metal orogastric tube (n= 7). One hr later, animals were killed by a blow to the head and their stomachs were removed and opened along the greater curvature, rinsed with saline and the area of gastric lesions was determined by computerized planimetry (Morphomat, Carl Zeiss, Germany), (30, 31) in which the margins of a wound as depicted on the digitized image are outlined on a computer screen and enclosed area is automatically determined by a suitable software algorithm. Computer-based planimetry of digital images can provide rapid, accurate and reliable estimates of wounded area as compared to other methods. The mean ulcer area is expressed in square millimeters (mm^2^).

***Drug treatment***

Neurotensin (Tocris Bioscience, UK) was dissolved in phosphate-buffered saline containing 0.5% (wt/vol) BSA and administered intravenously (i.v.) at doses of 1.5, 3, and 5 µM/kg (32), or intracerebroventricularly (i.c.v.) at doses of 0.5, 1, and 2.5 nM/rat (33), 30 min before the ethanol challenge (n= 7/group). In the case of gastroprotective activity induced by any dose of neurotensin, the CB_1_ receptor antagonist AM251 (Tocris Bioscience, UK) was dissolved in Tween 80 (Sigma Aldrich, Germany), dimethyl sulfoxide (Sigma Aldrich, Germany), and 0.9% saline (1:1:8) and injected i.c.v. at doses of 5, 10, and 15 nM/rat (34), 30 min before the administration of neurotensin (n= 7/group). If AM 251 could prevent neurotensin-induced gastroprotection at any dose tested, its effect was evaluated on either the intact stomach or ethanol-induced gastric lesions. Volume of injections in i.v. and i.c.v. routes were 5 ml/kg and 10 μl, respectively (35, 36).

***Surgical procedures***

A mixture of ketamine (80 mg/kg) and xylazine (10 mg/kg) was injected intrapertoneally during the surgery. For i.v. injections, PE 50 tubing filled with heparinized saline (100 U/ml) was implanted through the right femoral vein of each rat. For i.c.v. injections, each anaesthetized rat was placed in a stereotaxic apparatus. A burr hole was drilled through the skull 1.5 mm lateral to the midline and 1.5 mm posterior to the bregma on the right side. Through this hole, a 10 mm length of 20 gauge stainless steel hypodermic tubing was directed toward the right lateral ventricle. The cannula was lowered 4.2-4.5 mm below the surface of the skull perpendicularly and was fixed to the skull with acrylic cement. Animals were housed individually and allowed a 5-day recovery period before the experiments were initiated. I.c.v injections were given by hand to lightly restrained rats using a 50 µl Hamilton microsyringe over a period of 60 sec. At the end of the experiments, 5 μl of a methylene blue solution was injected into the cerebral ventricle through the cannula and the placement of the inner end of the cannula was verified for each rat. After decapitation, the brains were removed and sections were observed macroscopically to ascertain whether the cannula had been correctly placed into the lateral cerebral ventricle (36).

***Statistics***

Data were analysed by analysis of variance (ANOVA) followed by Tukey’s *post hoc* test. Results are expressed as mean±SEM (7 animals per group). The level of significance was set at *P< *0.05. 

## Results

***Effect of acidified ethanol on gastric mucosa***

**Figure 1:**
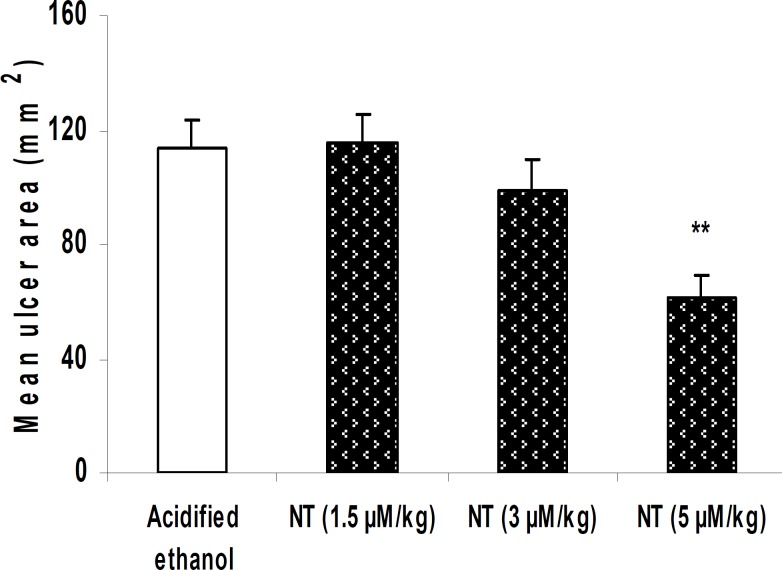
Effect of neurotensin (i.v.) on gastric mucosal injury induced by acidified ethanol. Mean area of gastric lesions induced by acidified ethanol was significantly reduced due to the pretreatment with 5 µM/kg neurotensin.

Rats given acidified ethanol developed mucosal damage in the oxyntic mucosa. Mucosal lesions consisted of elongated bands of necrosis with a mean area of 113±9.7 mm^2^ (Figure 1). 

***Effect of peripheral administration of neurotensin on gastric mucosal injury induced by acidified ethanol***

Pretreatment with neurotensin (5 µM/kg, i.v.) significantly reduced acidified ethanol-induced gastric lesions (Figure 1, *P <*0.01). Neurotensin at lower doses showed no effect (Figure 1, *P>*0.05). 

***Effect of central administration of neurotensin on gastric lesions induced by acidified ethanol***

**Figure 2:**
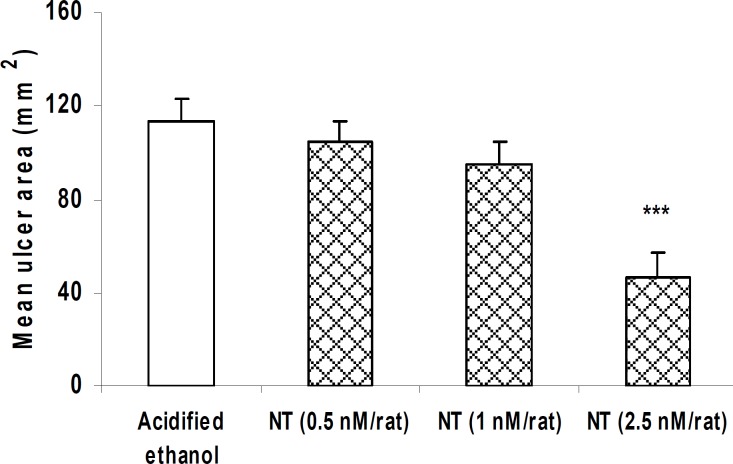
. Effect of neurotensin (i.c.v.) on gastric mucosal injury induced by acidified ethanol. As shown, pretreatment with neurotensin reduced gastric damage in a dose-dependent fashion.

Ethanol-induced ulcer area was significantly decreased due to i.c.v. pretreatment with 2.5 nM/rat neurotensin (Figure 2, *P <*0.001), while neurotensin at doses of 0.5 or 1 nM/rat did not alter the mean ulcer area (Figure 2, *P>*0.05).

***Effect of centrally injected CB***_1_ ***receptor antagonist on the gastroprotective effect of neurotensin***

**Figure 3:**
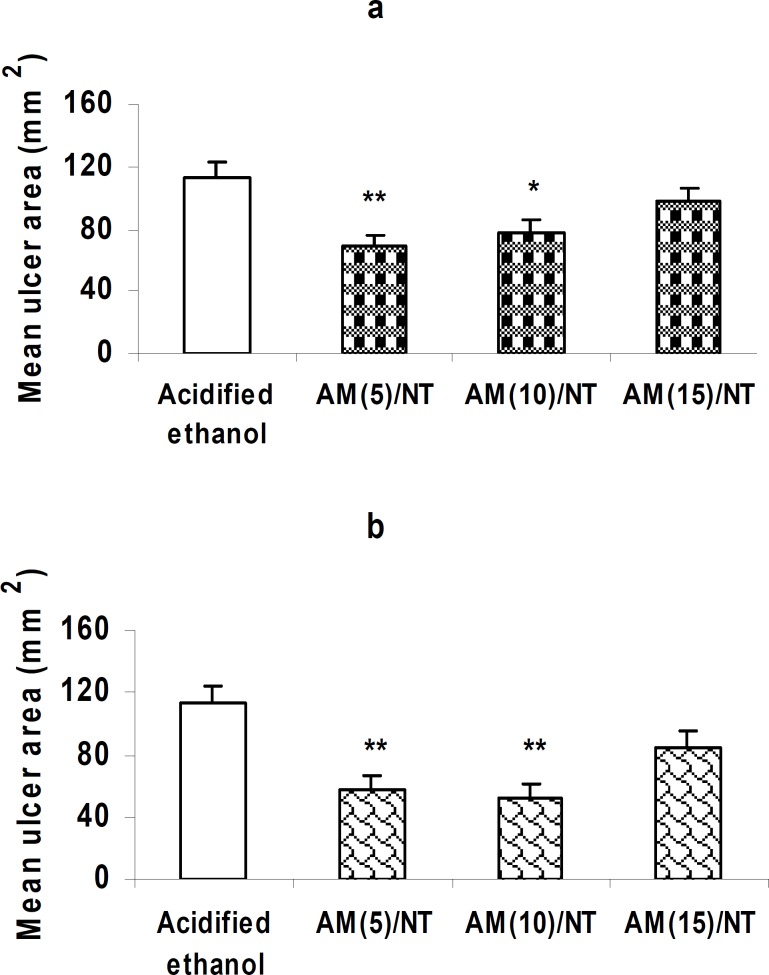
Effect of the CB_1_ receptor antagonist on the gastroprotection induced by neurotensin. Pre-application of AM 251 (15 nM/rat, i.c.v.) inhibited the gastroprotective effect of peripherally (5 µM/kg) or centrally (2.5 nM/rat) administered neurotensin (a and b, respectively).

The mean area of gastric lesions did not differ significantly between the acidified ethanol- or neurotensin-treated groups when AM 251 (15 nM/rat, i.c.v.) was injected prior to i.v. or i.c.v. administration of neurotensin (Figures 3a and 3b, respectively, *P>*0.05). Pretreatment with AM251 at doses of 5 or 10 nM/rat did not alter the significant reduction of gastric lesions induced by peripherally or centrally administered neurotensin (Figure 3a: *P <*0.01 and *P <*0.05; Figure 3b: *P <*0.01 and *P <*0.01, respectively).

Each column represents mean± SEM (n= 7/group). (NT: Neurotensin, µM: micromolar), ^**^P< 0.01 vs. acidified ethanol group

AM 251 at doses of 5 or 10 nM/rat did not alter cytoprotection induced by neurotensin. Each column represents mean±SEM (n= 7/group).{AM(5): AM251 (5 nM/rat), AM(10): AM251 (10 nM/rat), AM(15): AM251 (15 nM/rat), AM(...)/NT: Administration of AM251 before neurotensin treatment}.^*^P <0.05, ^**^P <0.01 vs. acidified ethanol group.

**Figure 4:**
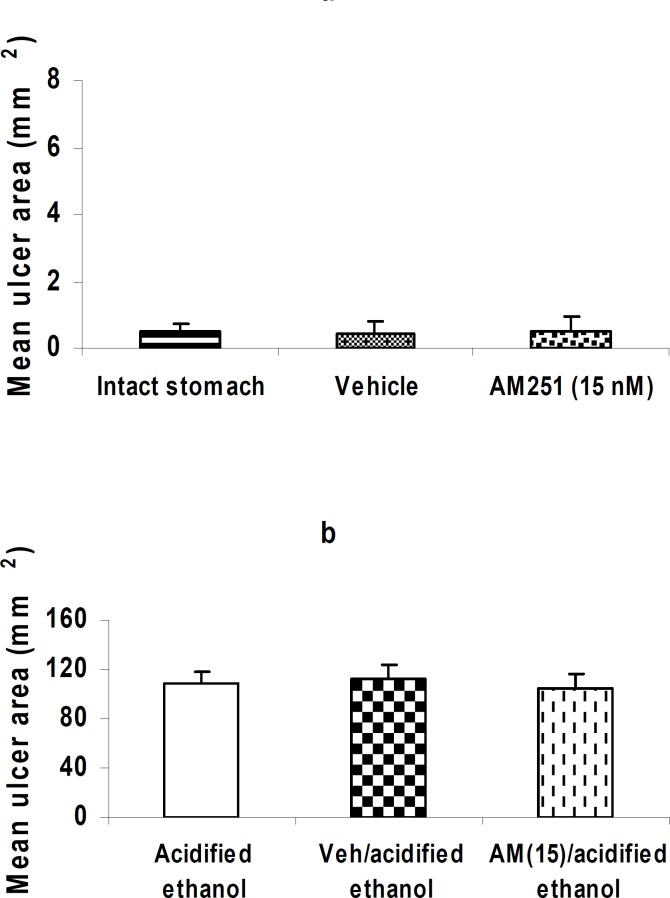
Effect of AM 251 on the intact stomach and gastric mucosal injury induced by acidified ethanol. Administration of AM 251 (15 nM/rat, i.c.v.) alone did not affect the mucosal integrity in the intact stomach (a, *P> *0.05).

Moreover, application of AM 251 (15 nM/rat, i.c.v.) before the induction of gastric lesions did not alter the mean ulcer area (b, *P> *0.05). Each column represents mean±SEM (n=7/group). (Vehicle: vehicle of AM251; Veh/acidified ethanol: injection of the vehicle of AM 251 thirty min before the ethanol challenge; AM (15)/acidified ethanol: injection of AM 251 (15 nM/rat, i.c.v.) 30 min before the ethanol challenge).

***Effect of AM251 on the intact stomach and gastric mucosal injury induced by acidified ethanol***

Administration of AM 251 (15 nM/rat, i.c.v.) alone did not affect the mucosal integrity in the intact stomach (a, *P>*0.05). Application of AM 251 (15 nM/rat, i.c.v.) before the induction of gastric lesions by acidified ethanol did not alter the mean ulcer area (b, *P> *0.05). 

## Discussion

In recent years, the therapeutic potential of neuropeptides in various health problems has attracted growing interest. In the present study, we used a model of gastric lesion in order to evaluate the gastroprotective potential of the gut-brain neuropeptide, neurotensin, either in peripheral or central application. We also investigated the possible implication of CB_1_ cannabinoid receptors in this regard. The mean ulcer area in rats given acidified ethanol indicated a development of gastric lesions (Figure 1). Based on the time interval between the induction of gastric mucosal damage and macroscopical evaluation of the lesions (1 hr), it appears that the mucosal damage is due to the direct action of acidified ethanol on the gastric mucosa. As shown in Figures 1 and 2, neurotensin given peripherally or centrally reduced acidified ethanol-induced gastric lesions in a dose-dependent fashion. These findings represent the gastroprotective activity of neurotensin as well as its potential implication in the healing process or tissue repair in the ulcerated mucosa of rat stomach induced by a corrosive substance such as acidified ethanol. Other neuropeptides have also been shown to exert protective effects against ethanol-induced mucosal lesions. For example, thyrotropin releasing hormone (TRH) when injected intracisternally or directly into the dorsal motor nucleus of vagus, reduced ethanol-induced mucosal lesions (10). Moreover, different opioid peptides including nociceptin, ghrelin and orexin have been shown to induce mucosal protection against ethanol-induced mucosal lesions (12, 13). 

Regarding the underlying mechanism(s) through which neurotensin exerts its gastroprotective effects, it has been previously postulated that neurotensin enhances restoration of epithelial integrity by increasing migration of epithelial cells into denuded regions of the gastrointestinal mucosa and elevates the release of protective prostaglandins (37). In addition, according to the involvement of vagal efferent nerves in the gastroprotection induced by adrenomedullin and TRH (14, 15), it is possible that neurotensin exerts its cytoprotective effect via the enhancement of parasympathetic outflow to the stomach. Interestingly, Nishikawa and colleagues have shown the healing effect of dopamine in the gastric ulcers (38). Since centrally administered neurotensin is able to release dopamine from mesolimbic terminal regions (37), therefore, dopaminergic neurotransmission may also be implicated in neurotensin-induced gastroprotection. 

As aforementioned, less attention has been previously paid to the central processes or mediators that may be involved in the maintenance of gastric mucosal defence mechanisms. Therefore, we evaluated the possible implication of central cannabinoid CB_1_ receptors in neurotensin-induced gastroprotection. These receptors are located in the brain areas which are implicated in the regulation of gastric functions (39). According to the previous reports, cannabinoids affect numerous GI functions such as the inhibition of gastric motility in rat or mice through the activation of CB_1_ receptors (40). Furthermore, cannabinoids have been shown to reduce formation of the experimental gastric ulcers. For example, in gastric ulceration induced by water immersion and restrain stress, anandamide has shown a protective effect (41). In a study conducted by Germano and co-workers, the synthetic cannabinoid WIN 55, 212-2 exhibited anti-ulcer effect in the cold/restraint stress model (42). In addition, ACEA (arachidonyl-2-chloroethylamide), the selective cannabinoid CB_1_ receptor agonist, significantly reduced the ulcer formation in aspirin-induced gastric ulcer (43). Our findings show that centrally administered CB_1_ receptor antagonist, AM 251, dose-dependently prevents the cytoprotective action of peripherally or centrally administered neurotensin (Figure 3, a and b), while, AM 251 shows no effect by itself (Figure 4, a and b). These findings suggest a critical role for the centrally located CB_1_ receptors in the gastroprotective effect of neurotensin. Meanwhile, the precise site of action of the centrally-initiated gastroprotection remains to be clarified. Since, the dorsal vagal complex is supposed to play an important role in the centrally-induced gastroprotection and the CB_1 _receptors are located in this area (28), therefore, it is reasonable to speculate that the site of action of the centrally-initiated gastroprotection is, at least in part, the dorsal vagal complex. Altogether, it appears that activation of the central CB_1_ receptors through the synthesis or release of endogenous neurotensin initiates a chain of events that results in the gastric protection against mucosal injury induced by acidified ethanol. This may serve as a basis for an interaction between the cannabinergic and neurotensinergic systems in gastric mucosal defence. Meanwhile, it should be taken into consideration that neuropeptide-mediated effects may be modulated by complex interactions with other mediators such as growth factors and cytokines which are involved in tissue restitution (44, 45).

## Conclusion

The results of this study provide evidences that peripherally or centrally given neurotensin exhibits a dose-dependent gastroprotection against damage caused by acidified ethanol. Considering a wide variety of effects mediated by neurotensinergic system, neurotensin may represent a potential therapeutic target in disorders associated with chronic mucosal ulcerations. Moreover, the gastroprotective action of neurotensin requires the activation of central CB_1_ receptors. This indicates the importance of centrally-initiated gastroprotection as well as the prominent role of the endocannabinoid system in this regard.
